# Soil Sample Preservation Strategy Affects the Microbial Community Structure

**DOI:** 10.1264/jsme2.ME20134

**Published:** 2021-02-10

**Authors:** Mariia Pavlovska, Ievgeniia Prekrasna, Ivan Parnikoza, Evgen Dykyi

**Affiliations:** 1 State Institution National Antarctic Scientific Center, 16 Taras Shevchenko Blvd., 01601, Kyiv, Ukraine; 2 National University of Life and Environmental Sciences of Ukraine, 15, Heroiv Oborony Str., 03041, Kyiv, Ukraine

**Keywords:** sample preservation, soil microbial communities, storage buffer, DESS

## Abstract

Sample preservation is a critical procedure in any research that relies on molecular tools and is conducted in remote areas. Sample preservation options include low and room temperature storage, which require freezing equipment and specific buffering solutions, respectively. The aim of the present study was to investigate whether DNA/RNA Shield 1x from Zymo Research and DESS (Dimethyl sulfoxide, Ethylenediamine tetraacetic acid, Saturated Salt) solution performed similarly to snap freezing in liquid nitrogen. Soil samples were stored for 1 month in each of the buffers and without any solution at a range of temperatures: –20, +4, and +23°C. All treatments were compared to the “optimal treatment”, namely, snap freezing in liquid nitrogen. The quality and quantity of DNA were analyzed, and the microbial community structure was investigated in all samples. The results obtained indicated that the quantity and integrity of DNA was preserved well in all samples; however, the taxonomic distribution was skewed in samples stored without any solution at ambient temperatures, particularly when analyses were performed at lower taxonomic levels. Although both solutions performed equally well, sequencing output and OTU numbers in DESS-treated samples were closer to those snap frozen with liquid nitrogen. Furthermore, DNA/RNA Shield-stored samples performed better for the preservation of rare taxa.

Molecular tools are rapidly being developed, which is providing more opportunities to study environmental microbial diversity and the broad spectrum of its adaptations. The resolution achieved by metagenomics is higher than that by culture-based techniques, which only allows ~1% of the total diversity to be examined (Hugerth and Andersson, 2017). Even though these technical developments have revolutionized the field of microbial ecology, there is one major drawback—they are strongly dependent on sample preservation strategies when immediate processing is not available. DNA and RNA degradation, which may occur in microbial samples under the influence of UV, high ambient temperatures, and multiple freeze-thaw cycles, may skew results, thereby preventing original community structure reconstruction (Lee *et al.*, 2019). In addition to nucleic acid degradation, the growth of specific taxa may occur under room temperature storage, whereas the proliferation of other community members will stop. It had previously been claimed that taxa capable of proliferation in samples stored at room temperature were not physiologically important; however, this was subsequently confuted (Amir *et al.*, 2017) and is not relevant to microbial ecology studies.

Freezing at –80°C is an ideal strategy for the preservation of microbial samples (Lee *et al.*, 2019). The distribution of microbial taxa remains constant and allows for a reliable quantitative analysis for at least 6 months upon sampling when samples are kept at –80°C (Carroll *et al.*, 2012). Moreover, high-quality DNA suitable for a 16S analysis was obtained from samples stored at –80°C for 14 years (Kia *et al.*, 2016). Snap freezing in liquid nitrogen is considered to be an even better option because it quickly terminates metabolic processes in bacterial cells, which is beneficial for subsequent metatranscriptomic and proteomic analyses (Fouhy *et al.*, 2015).

The main disadvantage of liquid nitrogen is the existence of multiple restrictions for its transportation. When –80°C and liquid nitrogen are not options, it is preferable to keep samples refrigerated (4°C), which limits bacterial growth and slows down metabolic processes, thereby allowing for a reliable metagenomic analysis within 24 h of collection (Vandeputte *et al.*, 2017).

However, refrigeration requires some facilities that are not always available in the field. It also relies on a constant energy supply and blackouts may lead to freeze-thaw and sample loss. Therefore, buffering solutions that enable room temperature storage are gaining popularity. This approach allows for study design flexibility and easier logistics, which is crucial for studies in remote areas.

Many options are currently available for the preservation of biological samples of varying origins and include FTA cards (Whatman, GE Healthcare), FTA Elute cards (Whatman, GE Healthcare), commercial solutions (RNAlater [Ambion], DNAguard [Biomatrica], DNA/RNA Shield 1x [Zymo Research]), and nonproprietary DESS solution (Gray *et al.*, 2012). These options differ according to the type of sample, the type of nucleic acid they preserve (some preserve both RNA and DNA, while others only preserve DNA), and the storage temperatures they require (some allow for room temperature storage, whereas others require cooling equipment for optimal performance).

The aim of the present study was to investigate which storage option is the best for preserving microbial DNA from soil. This issue is crucial for us because we are involved in Antarctic microbial studies, which strongly rely on an adequate sample preservation strategy. Commercial DNA/RNA Shield 1x from Zymo Research and DESS (Dimethyl sulfoxide, Ethylenediamine tetraacetic acid, Saturated Salt) solution (Lee *et al.*, 2019) were compared with snap freezing in liquid nitrogen.

## Materials and Methods

### Sample collection and preservation

Surface soil samples (20 g) were collected in August 2019 in an urban area of Kyiv (Ukraine) (50°26'29" N, 30°31'28" E) for the purpose of establishing an optimal sample storage protocol. The soil type was grey forest soil with pH 5.1±0.17 measured in a 1:5 suspension of soil in double-distilled water according to ISO 10390:2005 on a SevenMulti Benchtop Meter (Mettler-Toledo). The total organic carbon content constituted 52‍ ‍mg g^–1^ and was assessed by the wet combustion method (ISO 14235: 1998), whereas humus accounted for 1.57±0.27% measured according to the Turin method. The soil gravimetric water content constituted 6%.

Samples were homogenized using a sterile mortar and pestle for 5‍ ‍min and 1‍ ‍g of homogenized soil was transferred to 15-mL falcon tubes. All mortars and pestles were sterilized in an autoclave at 121°C for 20‍ ‍min. The experiment was designed such that 10 samples were divided into 3 groups: 1. stored without any solution, 2. stored with DNA/RNA Shield 1x, and 3. stored with DESS solution (Table 1). DESS solution was prepared as shown in Table S3. The control sample was immediately frozen in liquid nitrogen. DNA/RNA Shield was added to 3 tubes at a proportion specified by the manufacturer (1 sample: 9 DNA/RNA Shield). Similarly, DESS was added to the remaining 3 tubes at a proportion used by Yoder *et al.* (1 sample: 3 DESS).

Samples were stored at a range of temperatures: –180, –20, +4, and +23°C for one month until processed (Table 1). The rationale behind the choice of a 1-month storage period is that this is sufficient to transfer samples from the field in remote areas to the laboratory. The short-term storage periods of 11 and 14 days used in previous studies were selected (Lauber *et al.*, 2010; Brandt *et al.*, 2014). Refrigerating equipment is generally available for long-term storage. Molecular analyses were subsequently performed once for each treatment. DNA was extracted from all samples using the Quick-DNA Fecal/Soil Microbe Kit (Zymo Research) and according to the standard procedures described by the manufacturer. DNA quality and purity were assessed using a Nanodrop ND 1000 (Thermo Scientific) spectrophotometer (Table 2).

### PCR and sequencing

PCR was also performed to examine the presence of the 16S gene, which is routinely used as a marker for the taxonomic and phylogenetic assignments of prokaryotes. 16S V4 was amplified with S-D-Bact-0341-b-S-17 (5′-CCTACGGGNGGCWGCAG-3′) and S-D-Bact-0785-a-A-21 (5′-GACTACHVGGGTATCTAATCC-3′) primers (Klindworth *et al.*, 2013) using Mastercycler^®^ Gradient (Eppendorf). Each 25-μL reaction contained 2.5‍ ‍μL of 10xPCR Buffer without MgCl_2_ (Roche), 2.5‍ ‍μL of MgCl_2,_ 2.5‍ ‍μL of dNTPs, 1.5‍ ‍μL of a forward primer and 1.5‍ ‍μL of a reverse primer, 0.5‍ ‍μL of Taq polymerase, 13‍ ‍μL of ddH_2_O, and 1‍ ‍μL of sample DNA (Table S1). The PCR program was set as follows: denaturation at 95°C for 5‍ ‍min, 25 cycles of: denaturation at 95°C for 40‍ ‍s, annealing at 55°C for 2‍ ‍min, and elongation at 72°C for 2‍ ‍min; followed by a final elongation at 72°C for 7‍ ‍min (Table S2). All reactions were run in triplicate alongside the negative controls containing all components of the reaction mix, except the DNA template. The presence and concentration of amplicons (464 bp) were analyzed using gel electrophoresis (Fig. S1).

The concentration of DNA and presence of the target amplicon do not necessarily indicate the suitability of a storage method. Therefore, the taxonomic structure and diversity microbial communities were examined via the Illumina MiSeq sequencing of the 16S V4 region (Molecular Research LP, Mr. DNA). Bacterial diversity in samples was evaluated using the MiSeq 16S rRNA protocol based on the bTEFAP process (Dowd *et al.*, 2008; Chiodini *et al.*, 2015) with the 16S rRNA gene V4 region bacterial primer pair S-D-Bact-0341-b-S-17 and S-D-Bact-0785-a-A-21 (Klindworth *et al.*, 2013) with a barcode on the forward primer. The conditions of single-step 28 cycle PCR using the HotStarTaq Plus Master Mix Kit (Qiagen) were as follows: 94°C for 3‍ ‍min, followed by 28 cycles of 94°C for 30‍ ‍s, 53°C for 40‍ ‍s, and 72°C for 1‍ ‍min, with the final elongation step at 72°C for 5‍ ‍min. All reactions were run in triplicate with negative controls. Amplification success was examined via 2% agarose gel electrophoresis of PCR products. Multiple samples were pooled together in equal proportions based on their molecular weight and DNA concentrations, followed by the purification of pooled samples with calibrated Ampure XP beads. The resulting pooled and purified PCR product was subsequently used to prepare the Illumina DNA library. Sequences were deposited in the NCBI Sequence Read Archive (PRJNA625500).

### Bioinformatics and statistical analysis

Raw sequencing data were demultiplexed, denoised, quality filtered, and taxonomically assigned using the QIIME2 2019.7 pipeline (Bolyen, E., Rideout, J.R., Dillon, M.R., Bokulich, N.A., Abnet, C., Al-Ghalith, G.A., *et al.* (2018) QIIME 2: Reproducible, interactive, scalable, and extensible microbiome data science. *PeerJ Preprints* https://doi.org/10.7287/peerj.preprints.27295v2). Sequences shorter than 150 bp were discarded. After quality filtering, there were between 89,891 and 105,863 sequences per sample with a total of 63,089–76,931 operational taxonomic units (OTUs) (Table S4 and S5). Taxonomy was assigned according to the Greengenes 13_8 database. The Shannon index and Bray-Curtis distance matrix were estimated with the “diversity core metrics” plugin in QIIME2 2019.7.

Statistical analyses were conducted using Origin 2019 (OriginLab Corporation). The Shapiro-Wilks test indicated a non-normal distribution of taxonomic data. Therefore, the significance of differences between sample storage treatments was estimated using the Kruskal-Wallis test at the class, family, and genus levels. Spearman’s correlation analysis was performed to estimate the relationship between optimal storage at –180°C and other treatments.

## Results

DNA concentrations varied in the range of 12.7–53.3‍ ‍ng‍ ‍μL^–1^ and did not correlate with either the preservation method or temperature treatment. The lowest concentration was observed in the sample treated with DESS and stored at –‍20°C. The concentration of DNA and purity 260/280 ratio was sufficiently high to allow for a downstream 16S analysis in all samples analyzed (Pichler *et al.*, 2018) (Table 2).

Gel electrophoresis identified the presence of the target 16S 464-bp fragment in all samples (Fig. S1).

Sequencing output yielded between 89891 and 105863 sequences (Table S4) of the partial 16S rRNA gene per sample with an average length of 251 bp. The highest number of sequences was detected in the sample stored at –180°C without any buffer; however, the DESS-stored sample had a similar sequencing output of 105,823 when stored at –20°C. Rarefaction curves indicated that deeper sequencing would not have resulted in significantly higher estimates, which suggests that both abundant and rare taxa were covered (Fig. S2).

There were 63,089–76,931 OTUs detected in the communities, with the highest number being observed in samples stored at –180°C (Table S5). Both buffers resulted in a better storage capacity than the “no-buffer” treatment, particularly at room temperature. The number of OTUs detected in DESS-stored samples at –20°C was the closest to the optimal treatment (–180°C treatment), reaching 75,437.

**Fig. 1.**
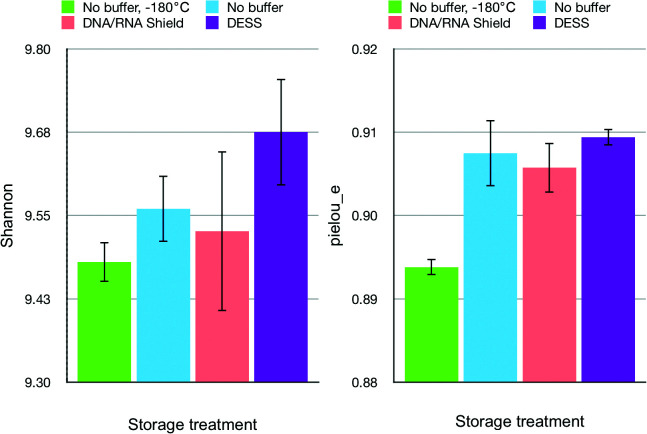
Microbial community (a) richness and (b) evenness in differentially treated samples

Richness estimated with the Shannon index and evenness indicated no significant differences between differentially-stored samples (Fig. 1). Moreover, the DESS-treated sample was characterized by higher richness than the sample stored at –180°C without any buffer. This may have been the result of a lower DNA yield from that sample. However, based on the high number of OTUs in samples stored at –180°C, we suggest that the discrepancy between the OTU number and richness estimates was due to the Shannon index scaling richness based on community evenness. In this case, samples stored at –180°C may be characterized by the stronger dominance of a few taxonomic groups with a higher read number than other treatments.

**Fig. 2.**
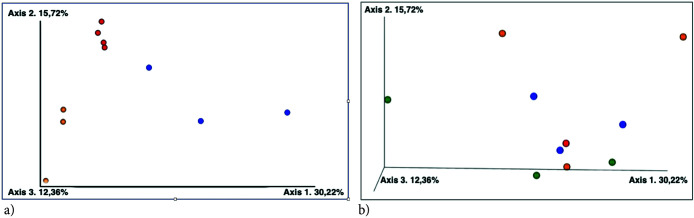
UniFrac estimations of biological distances between microbial communities in different sample treatments: (a) based on the storage buffer factor: red—no buffer, orange—DNA/RNA Shield, blue—DESS; (b) based on the storage temperature factor: red— –180°C, blue— –20°C, orange—+4°C, green—+23°C

The UniFrac distance matrix (Fig. 2) showed that storage buffer plays a more important role in shaping differences between communities than the temperature factor. Samples stored without any buffer clustered together with the closest distance between the –180 and –20°C treatments. At the same time, DNA/RNA Shield- and DESS-stored samples were both characterized by certain variations.

A microbial taxonomic analysis was performed at the class, family, and genus levels for the purpose of identifying the taxa causing this variability. Twenty-eight classes, 20 families, and 34 genera were detected after the 0.5% taxa relative abundance cut-off had been applied to all samples.

**Fig. 3.**
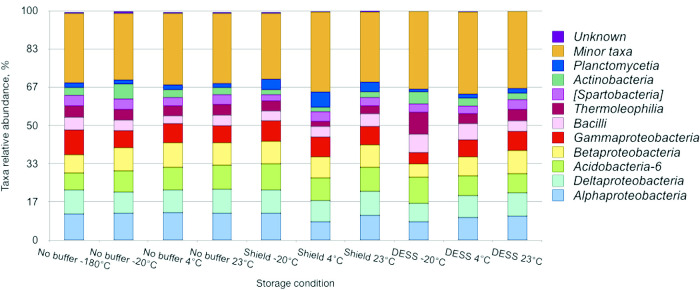
Relative abundance of bacterial classes in soil samples stored under different conditions

The top 10 bacterial classes were shared between differentially treated samples with *Alphaproteobacteria*, *Deltaproteobacteria*, *Acidobacteria*, *Betaproteobacteria*, and *Gammaproteobacteria* comprising approximately half of the community (Fig. 3). DESS and DNA/RNA Shield-treated samples were both characterized by greater variations in the distribution of taxa than samples stored at –‍180°C. *Planctomycetia* was overrepresented in all DNA/RNA Shield-treated samples, whereas DESS-treated samples stored at –20°C had an excess of *Thermoleophilla* and *Actinobacteria* at the cost of *Gammaproteobacteria*. However, the Kruskal-Wallis test did not identify any significant differences in the distribution of taxa between buffer-treated samples and non-treated –180 and –20°C samples. At the same time, slight differences were observed in *Alphaproteobacteria*, *Cytophagia*, *Chloracidobacteria*, and *MB-A2-108* ratios (H=3.88, *P*=0.05) when two storage buffers were compared (Table S6).

**Fig. 4.**
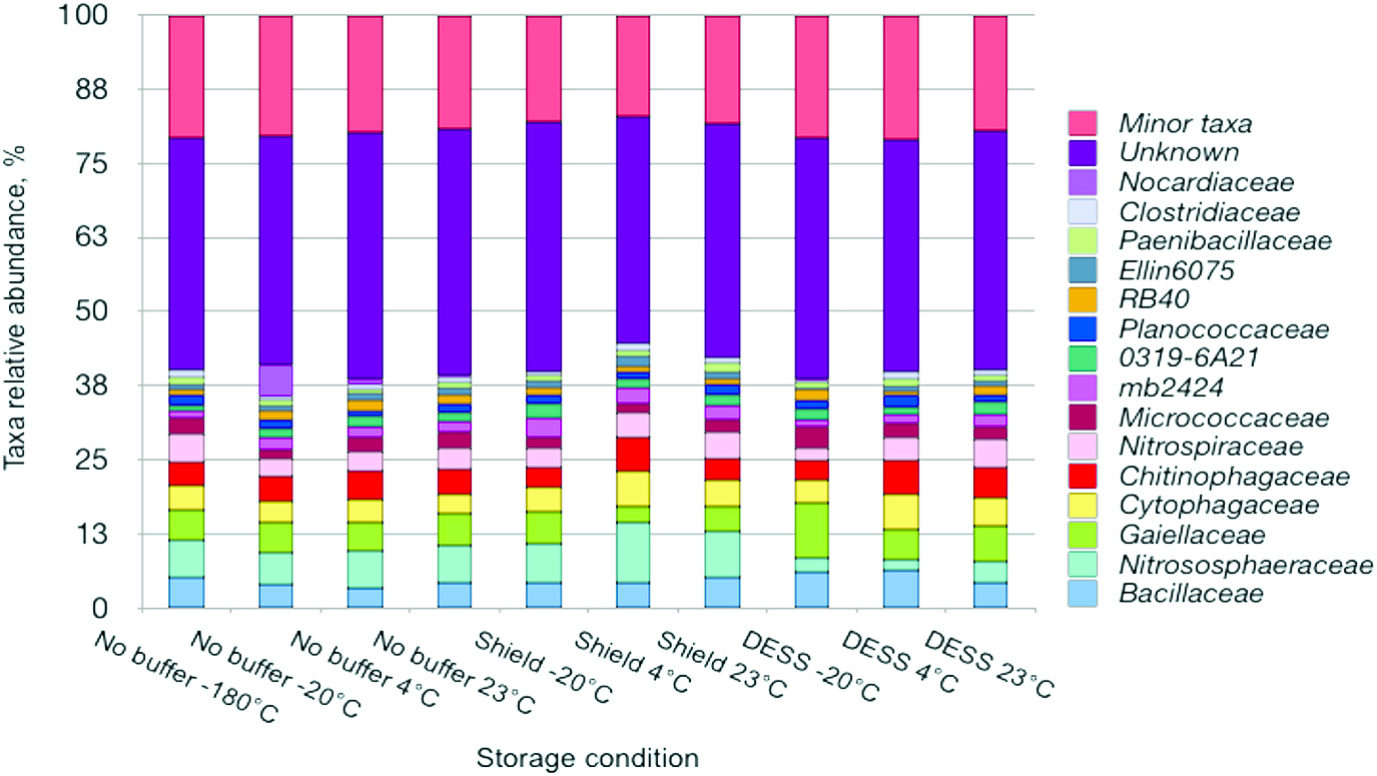
Relative abundance of bacterial families in soil samples stored under different conditions

In total, 40% of taxa failed to be identified at the family level and this ratio was equal between differentially stored samples. *Bacillaceae*, *Nitrososphaeracea*, *Gaiellaceae*, *Cytophagaceae*, *Chitinophagaceae*, and *Nitrospiraceae* represented more than 25% of the microbial community in all samples (Fig. 4). The microbial community of samples stored without any buffer at –20, 4, and 23°C differed from those stored at –180°C because it harbored less *Nitrospiraceae* and significantly more *Nocardiaceae*. The taxonomic distribution in DESS-treated samples stored at –‍20°C was unique due to the ratio of some dominant taxa—*Nitrososphaeracea* and *Gaiellaceae*. The remaining DESS-stored samples were similar to untreated samples stored at –‍180°C. However, these differences between DESS-treated and untreated samples were not significant when the Kruskal-Wallis test was performed. Highly abundant *Nitrososphaeraceae*, *Cytophagaceae*, and *mb2424* and rare *RB40*, *Nocardiaceae*, *EB1017*, and *koll13* contributed to the discrepancy between DNA/RNA Shield-stored and –180°C-stored samples according to the Kruskal-Wallis test (H=3.88, *P*=0.05, Table S7). Similarly, the ratio of *Nitrososphaeraceae*, *koll13*, and *Nocardiaceae* differed between the samples kept in two storage buffers (H=3.88, *P*=0.05, Table S8).

**Fig. 6.**
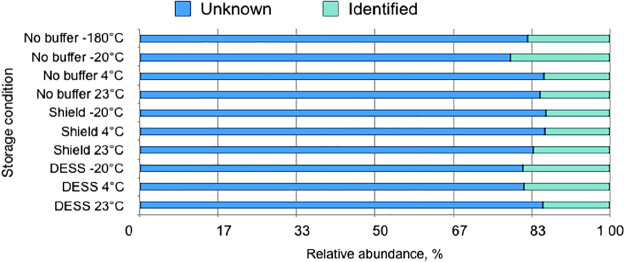
Ratio of taxa not identified at the genus level

The share of taxa failed to be identified at the genus level comprising 83% on average, with the highest being in buffer-free samples stored at 4 and 23°C and in all DNA/RNA Shield-treated samples (Fig. 6). This may be putatively explained by DNA degradation, which reduced DNA quality and resulted in sequencing errors (Krehenwinkel *et al.*, 2018). The highest number of genera was detected in DESS-treated samples stored at –20°C (32) and in those stored at –180°C (31), whereas untreated samples stored at –20, +4, and 23°C were characterized by the lowest diversity harboring 28, 23, and 28 genera, respectively.

**Fig. 5.**
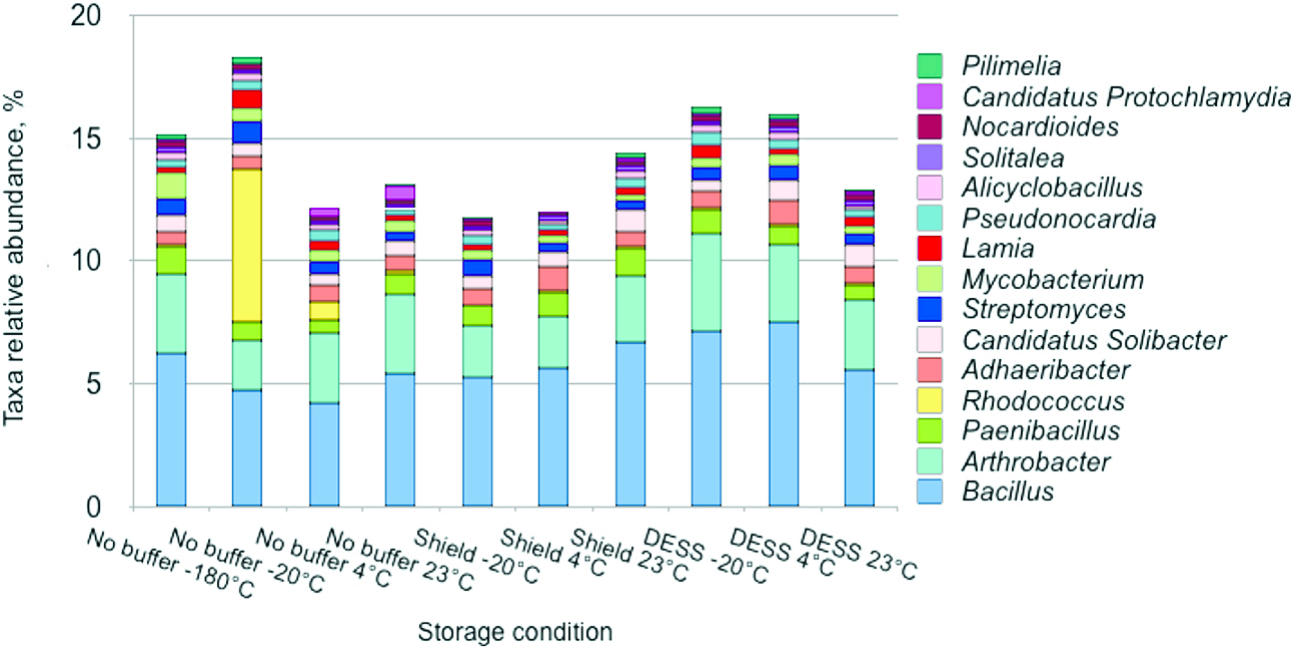
Relative abundance of bacterial genera in soil samples stored under different conditions

The majority of taxa identified at the genus level were represented by *Bacillus* and *Arthrobacter*, the ratio of which varied between treatments (Fig. 5). Samples stored at –20 and +4°C were unique and this was mainly due to the presence of the genus *Rhodococcus*. DESS-treated samples were the closest to those stored at –180°C in terms of the relative abundance of genera; however, *Mycobacterium* was underrepresented in all samples.

Spearman’s correlation was performed at the class, family, and genus levels in order to quantitively analyze the taxonomic structure similarity of differentially treated samples (Table S9, S10, and S11). As expected, all relationships were significant, but generally became weaker when lower taxonomic levels (*e.g.*, genus) were analyzed. The taxonomic structure of most samples strongly correlated (ρ>0.9, *P*<0.05) with the distribution of taxa in samples stored at –‍180°C. The lowest coefficients were for +4°C storage in DNA/RNA Shield and –20°C in DESS, whereas all samples stored without any buffer performed equally well independent of the storage temperature.

The correlation pattern changed when diversity was analyzed at the family level. Samples stored without any buffer had a weaker correlation with those stored at –180°C in comparisons with soil kept in DNA/RNA Shield (ρ>0.92, *P*<0.05) and DESS (ρ>0.86, *P*<0.05).

The majority of correlation coefficients were lower than 0.8 when bacterial diversity was analyzed at the genus level. However, samples stored in both buffers were similar to soil stored at –180°C in terms of their taxonomic structure (ρ>0.7, *P*<0.05), in contrast to samples kept without any preserving solution. It is important to note that the taxonomic structure was better preserved in samples kept at lower temperatures than in those stored at +4°C.

## Discussion

Adequate sample preservation is a critical point in any protocol because it is the first step and subsequent analysis quality is highly dependent on it. The issue of DNA and RNA storage in various types of samples becomes even more complicated when research is conducted in remote areas. However, remote regions represent a perfect natural laboratory to study biodiversity and its unique adaptations. Therefore, this research field is worth investigating, particularly for the development of optimal protocols. Since the transfer of samples from the field to the laboratory may require a significant amount of time, it is crucial to have a validated solution with performance tested in multiple treatments at various temperature regimes.

Therefore, we investigated the following storage strategies: 1. –180°C without any buffer, as the optimal treatment, 2. –20, +4, and 23°C without any buffer, 3. commercial DNA/RNA Shield from Zymo Research at –20, +4, and 23°C, 4. DESS at –20, +4, and 23°C. The storage period was 1 month.

DESS solution was included in the present study because it is highly affordable and easily made in any laboratory with basic chemicals and minimal resources, and, thus, is a potentially good low-cost option. This solution is suitable for room temperature storage for a period of up to 3 months (Gray *et al.*, 2012). DESS solution has already been used for a range of samples, and was previously shown to be superior to ethanol for coral DNA preservation (Gaither *et al.*, 2011) and also preserved bacterial DNA for up to 4.5 months (May *et al.*, 2011). A recent study reported that the fungal community structure was preserved better with DESS than with glycerol or PBS (Lee *et al.*, 2019).

According to the present results, all treatments performed equally well at preserving the overall quantity and integrity of DNA. Some variations were observed in the concentration and quality of DNA in samples stored under different temperature treatments; however, no correlations were observed between the temperature and storage method and the resulting DNA characteristics were elucidated. Since soil samples stored at room temperature without any buffer gave a similar DNA yield and quality, 1 month does not appear to be sufficient to allow for significant DNA degradation, even under the influence of UV and 23°C. However, the taxonomic analysis revealed differences in the bacterial community structure between differentially treated samples. Although the most abundant class distribution was similar between communities, differences became more obvious when lower taxonomic levels were analyzed. This discrepancy indicates that the concentration and quality of DNA do not provide adequate data for selecting the best sample storage method, which instead needs to be supported by a taxonomic analysis of the communities being studied.

Both storage buffers performed better than untreated samples when diversity was investigated at the genus level. Bacterial communities stored at ambient temperature without any protecting solution had a skewed taxonomic distribution and markedly lower taxa number with more OTUs that failed to be identified. These results were also supported by the weaker correlation between these samples and those stored at –180°C.

Our results differ from previous findings. Lauber *et al.* did not detect any influence of the ambient storage temperature on the composition of microbial communities. However, the storage period analyzed in that study was 14 days, which was twice as short as that in the present study and may be the cause of the discrepancy observed between their findings and the present results. Brandt *et al.* (2014) also did not detect any effect of the storage temperature on the composition of soil microbial communities stored for 11 days. Additionally, Lauber *et al.* did not analyze microbial diversity at the genus level, whereas we detected the greatest variation between differentially stored samples when genus-level diversity was examined. Similar to Lauber *et al.*, Tatangelo *et al.* (2014) did not detect significant degradation between samples stored at +4 and +30°C and those frozen at –20°C. However, they identified a discrepancy in the microbial taxonomic structure induced by LifeGuard storage buffer. In contrast, samples stored with DESS solution did not significantly differ from frozen samples. This is consistent with some of the present results because we also detected an effect caused by storage buffer.

It is important to note that no optimal storage solution will perform in an identical manner to –180°C-stored samples. This was particularly evident when the distribution of less abundant taxa was analyzed. DNA/RNA Shield preserved some rare taxa (*e.g.*, *the* genus *Pilimelia*), but also overrepresented the *Planctomycetia* class. DESS solution performed better than DNA/RNA Shield at low temperatures (–20 and +4°C), but worse at 23°C. Both buffers failed to preserve *Mycobacterium* at a similar abundance to the –‍180°C treatment.

Therefore, the use of protecting buffer still appears to induce some variations in the sample taxonomic structure and highlights the necessity of replicates. Semi-quantitative data on the bacterial community taxonomic structure need to be treated with caution when optimal storage conditions are not available. One option to normalize these data is to perform quantitative RT-PCR for the purpose of absolute 16S gene quantification. The number of replicates will also contribute to data precision.

Nevertheless, the present results indicated that DNA/RNA Shield and DESS storage buffers both significantly improved microbial soil community preservation and may even be used at a high ambient temperature (+23°C) at least for a 1-month storage period when no –180°C option is available. Qualitative data on taxonomic diversity obtained from these samples are reliable at the class, family, and genus levels.

Our results indicate that DESS and DNA/RNA Shield are both suitable for soil sample preservation; however, the performance of each buffer is dependent on the chemical and physical characteristics of samples and, thus, need to be tested individually for each sample type (Rissanen *et al.*, 2010).

## Conclusions

Sample preservation is a critical point in any research, particularly that conducted in remote areas, because it significantly influences any downstream analyses. Therefore, we tested several sample storage strategies. Soil samples were preserved for 1 month with two storage buffers (DNA/RNA Shield from Zymo Research and nonproprietary DESS solution) within a range of storage temperatures (–180, –20, +4, and +23°C). Our results indicate that both storage buffers performed equally better than the no-buffer treatment. The quantity and integrity of DNA were preserved in all samples. However, there were some discrepancies in the microbial taxonomic structure at the genus level from the –180°C treatment. Nevertheless, we recommend using either DNA/RNA or DESS solution when there is no –180°C (or –80°C) storage option. Qualitative data on microbial taxonomic diversity obtained from these samples are reliable at the class, family and genus levels. Further research needs to focus on examining the storage buffer capacity for community functional profile (RNA) preservation and the influence of freeze-thaw cycles on buffer performance.

## Author contributions

Mariia Pavlovska: Writing—Original draft preparation, Methodology, Investigation. Ievgeniia Prekrasna: Conceptualization, Formal Analysis, Investigation. Ivan Parnikoza: Supervision, Validation, Writing—Review & Editing. Evgen Dykyi: Writing—Review & Editing, Project Administration, Resources.

## Citation

Pavlovska, M., Prekrasna, I., Parnikoza, I., and Dykyi, E. (2021) Soil Sample Preservation Strategy Affects the Microbial Community Structure. *Microbes Environ ***36**: ME20134.

https://doi.org/10.1264/jsme2.ME20134

## Supplementary Material

Supplementary Material

## Figures and Tables

**Table 1.** Sample storage experiment design

Temperature, °C Storage buffer	+23	+4	–20	–180
No storage buffer	1‍ ‍g of sample	1‍ ‍g of sample	1‍ ‍g of sample	1‍ ‍g of sample
DESS	1V of sample+3V of DESS	1V of sample+3V of DESS	1V of sample+3V of DESS	
DNA/RNA Shield	1V of sample+9V of Shield	1V of sample+9V of Shield	1V of sample+9V of Shield	

**Table 2.** DNA quality and quantity estimated with the Nanodrop ND 1000 (Thermo Scientific) spectrophotometer

**#**	**Storage buffer**	**Temperature, °C**	**Average DNA concentration, ng μL^–1^**	**SD**	**260/280**	**260/230**
**0**	**—**	**–180**	26.79	0.45	1.72	0.86
**0.1**	**—**	**–20**	47.55	4.15	1.87	1.22
**0.2**	**—**	**4**	41.04	1.19	1.89	1.15
**0.3**	**—**	**23**	31.54	2.13	1.87	1.21
**1.1**	**DNA/RNA Shield**	**–20**	37.63	0.12	1.86	1.12
**1.2**	**DNA/RNA Shield**	**4**	53.34	7.56	1.74	0.28
**1.3**	**DNA/RNA Shield**	**23**	43.16	0.56	1.83	1.38
**2.1**	**DESS**	**–20**	12.70	0.53	1.77	0.63
**2.2**	**DESS**	**4**	31.39	2.37	1.61	1.03
**2.3**	**DESS**	**23**	32.69	0.89	1.81	0.93

## References

[B1] Amir, A., McDonald, D., Navas-Molina, J.A., Debelius, J., Morton, J.T., Hyde, E., et al. (2017) Correcting for microbial blooms in fecal samples during room-temperature shipping. mSystems 2: e00199-16. 28289733 10.1128/mSystems.00199-16 PMC5340865

[B2] Brandt, F.B., Breidenbach, B., Brenzinger, K., and Conrad, R. (2014) Impact of short-term storage temperature on determination of microbial community composition and abundance in aerated forest soil and anoxic pond sediment samples. Syst Appl Microbiol 37: 570–577. 25466922 10.1016/j.syapm.2014.10.007

[B3] Carroll, I.M., Ringel-Kulka, T., Siddle, J.P., Klaenhammer, T.R., and Ringel, Y. (2012) Characterization of the fecal microbiota using high-throughput sequencing reveals a stable microbial community during storage. PLoS One 7: e46953. 23071673 10.1371/journal.pone.0046953 PMC3465312

[B4] Chiodini, R.J., Dowd, S.E., Chamberlin, W.M., Galandiuk, S., Davis, B., and Glassing, A. (2015) Microbial population differentials between mucosal and submucosal intestinal tissues in advanced Crohn’s disease of the ileum. PLoS One 10: e0134382. 26222621 10.1371/journal.pone.0134382 PMC4519195

[B5] Dowd, S.E., Sun, Y., Wolcott, R.D., Domingo, A., and Carroll, J.A. (2008) Bacterial tag-encoded FLX amplicon pyrosequencing (bTEFAP) for microbiome studies: bacterial diversity in the ileum of newly weaned Salmonella-infected pigs. Foodborne Pathog Dis 5: 459–472. 18713063 10.1089/fpd.2008.0107

[B6] Fouhy, F., Deane, J., Rea, M.C., O’Sullivan, Ó., Ross, R.P., O’Callaghan, G., et al. (2015) The effects of freezing on fecal microbiota as determined using MiSeq sequencing and culture-based investigations. PLoS One 10: e0119355. 25748176 10.1371/journal.pone.0119355 PMC4352061

[B7] Gaither, M.R., Szabo, Z., Crepeau, M.W., Bird, C.E., and Toonen, R.J. (2011) Preservation of corals in salt-saturated DMSO buffer is superior to ethanol for PCR experiments. Coral Reefs 30: 329–333.

[B8] Gray, M.A., Pratte, Z.A., and Kellogg, C.A. (2012) Comparison of DNA preservation methods for environmental bacterial community samples. FEMS Microbiol Ecol 83: 468–477. 22974342 10.1111/1574-6941.12008

[B9] Hugerth, L.W., and Andersson, A.F. (2017) Analysing microbial community composition through amplicon sequencing: From sampling to hypothesis testing. Front Microbiol 8: 1561. 28928718 10.3389/fmicb.2017.01561 PMC5591341

[B10] Kia, E., Wagner Mackenzie, B., Middleton, D., Lau, A., Waite, D.W., Lewis, G., et al. (2016) Integrity of the human faecal microbiota following long-term sample storage. PLoS One 11: e0163666. 27701448 10.1371/journal.pone.0163666 PMC5049846

[B11] Klindworth, A., Pruesse, E., Schweer, T., Peplies, J., Quast, C., Horn, M., and Glöckner, F.O. (2013) Evaluation of general 16S ribosomal RNA gene PCR primers for classical and next-generation sequencing-based diversity studies. Nucleic Acids Res 41: e1. 22933715 10.1093/nar/gks808 PMC3592464

[B12] Krehenwinkel, H., Fong, M., Kennedy, S., Huang, E.G., Suzuki, N., Cayetano, L., and Gillespie, R. (2018) The effect of DNA degradation bias in passive sampling devices on metabarcoding studies of arthropod communities and their associated microbiota. PLoS One 13: e0189188. 29304124 10.1371/journal.pone.0189188 PMC5755739

[B13] Lauber, C.L., Zhou, N., Gordon, J.I., Knight, R., and Fierer, N. (2010) Effect of storage conditions on the assessment of bacterial community structure in soil and human-associated samples. FEMS Microbiol Lett 307: 80–86. 20412303 10.1111/j.1574-6968.2010.01965.x PMC3148093

[B14] Lee, K.M., Adams, M., and Klassen, J.L. (2019) Evaluation of DESS as a storage medium for microbial community analysis. PeerJ 7: e6414. 30740279 10.7717/peerj.6414 PMC6368006

[B15] May, L.A., Higgins, J.L., and Woodley, C.M. (2011) *Saline-Saturated DMSO-EDTA as a Storage Medium for Microbial DNA Analysis from Coral Mucus Swab Samples*. NOAA Technical Memorandum NOS NCCOS 127 and CRCP 15. Charleston, SC: NOAA/National Centers for Coastal Ocean Science.

[B16] Pichler, M., Coskun, Ö.K., Ortega-Arbulú, A.S., Conci, N., Wörheide, G., Vargas, S., and Orsi, W.D. (2018) A 16S rRNA gene sequencing and analysis protocol for the Illumina MiniSeq platform. MicrobiologyOpen 7: e611. 10.1002/mbo3.611 PMC6291791 29575567

[B17] Rissanen, A.J., Kurhela, E., Aho, T., Oittinen, T., and Tiirola, M. (2010) Storage of environmental samples for guaranteeing nucleic acid yields for molecular microbiological studies. Appl Microbiol Biotechnol 88: 977–984. 20730531 10.1007/s00253-010-2838-2

[B18] Tatangelo, V., Franzetti, A., Gandolfi, I., Bestetti, G., and Ambrosini, R. (2014) Effect of preservation method on the assessment of bacterial community structure in soil and water samples. FEMS Microbiol Lett 356: 32–38. 24840085 10.1111/1574-6968.12475

[B19] Vandeputte, D., Tito, R.Y., Vanleeuwen, R., Falony, G., and Raes, J. (2017) Practical considerations for large-scale gut microbiome studies. FEMS Microbiol Rev 41: S154–S167. 28830090 10.1093/femsre/fux027 PMC7207147

