# Supplementary material for: Soil Sample Preservation Strategy Affects the Microbial Community Structure
Source: Microbes Environ. 2021 Feb 10;36(1):ME20134. doi: 10.1264/jsme2.ME20134 (PMC7966943; doi:10.1264/jsme2.ME20134)
Supplement: Supplementary file 1 — Supplementary Material [file 36_20134_s1.pdf]

Supplementary data

Reaction component	Volume, µl
**10xPCR Buffer without $MgCl_2$ (Roche)**	2.5
**$MgCl_2$**	2.5
**dNTPs**	2.5
**Forward primer**	1.5
**Reverse primer**	1.5
**ddH_2O**	13
**Taq polymerase (manufacturer)**	0.5
**DNA**	1
**Total**	25

Table S1. PCR reaction for 16S V4 fragment detection

Step	Temperature, °C	Duration	Cycles
**Denaturation**	95	5 min	-
**Denaturation**	95	40 sec	25
**Annealing**	55	2 min	
**Elongation**	72	2 min	
**Final elongation**	72	7 min	-

Table S2. PCR program 16S V4 fragment detection

Component	Volume/weight
EDTA (MW: 372.2 g)	93.06 g
NaOH, 20%	60 ml
HCl, 25%	20 ml
DMSO	40 ml
NaCl	variable
ddH_2O	~1000 ml

Table S3. DESS solution recipe to get 1L of solution

DESS preparation procedure:

1. Add 93.06 g of EDTA (MW: 372.24 g) to the glass beaker
2. Add 200 ml of ddH_2O to get 0.25 M EDTA solution. Heat the solution until EDTA dissolves (t=30°C).
3. EDTA dissolving can be facilitated by adding 60 ml of 20% NaOH, which will increase the solution pH to 13.
4. Add ~20 ml of 25%HCl in order to decrease the solution pH to 7.5.
5. Add ddH_2O to adjust the volume to 800 ml.
6. Mix 40 ml of DMSO and 160 ml of ddH_2O and add to the solution.
7. Add NaCl until the solution becomes oversaturated (NaCl precipitates).
8. Autoclave the solution for 20 min at 121°C

	No buffer	DESS	Shield
**23C**	92197	94080	103919

**4C**	89891	91243	98888
**-20C**	97390	105823	94985
**-180C**	105863		

Table S4. Number of sequences in analyzed samples

	No buffer	DESS	Shield
**23C**	64769	66943	74036
**4C**	63407	63089	69049
**-20C**	70921	75437	66270
**-180C**	76931		

Table S5. Number of OTUs in analyzed samples

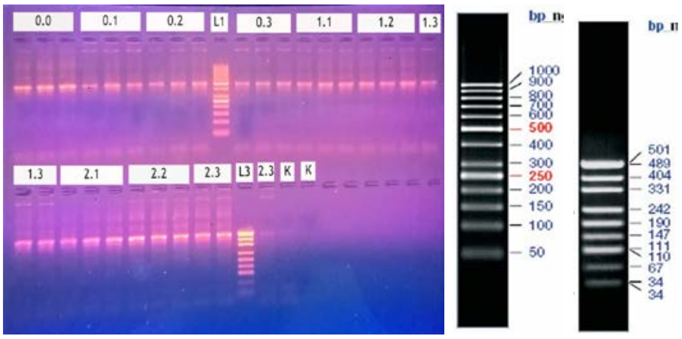

Figure S1. The presence of target 16S V3-V4 fragment in the samples analyzed. 0.X – without any storage buffer, 1.X – with DNA/RNA Shield (Zymo), 2.X – with DESS. X.1 - -20°C, X.2 - +4°C, X.3 - +23°C. L1 - GeneRuler 50 bp DNA Ladder (Thermo Fisher Scientific, USA), L3 - pUC19 DNA/MspI (HpaII) Marker (Thermo Fisher Scientific, USA), K – negative controls.

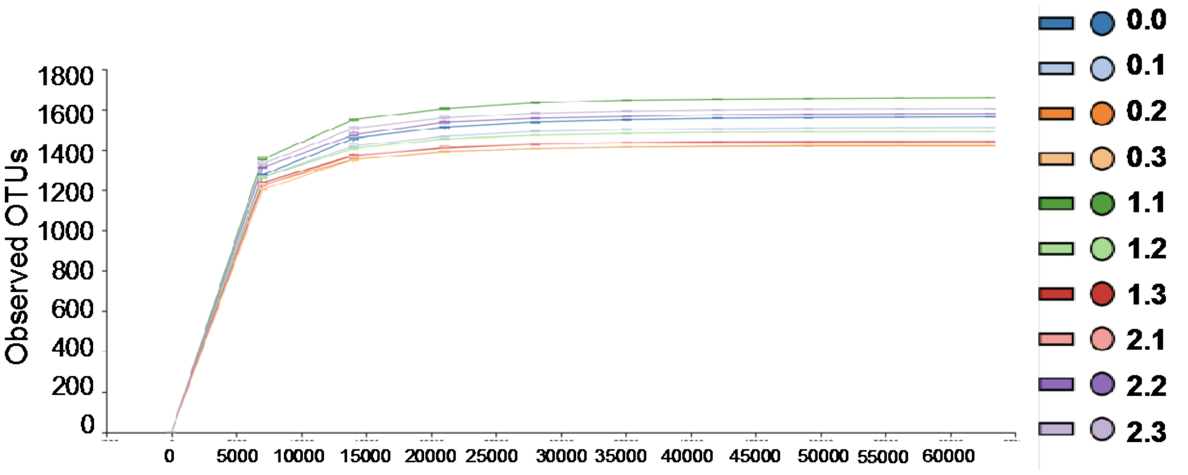

Figure S2. Rarefaction curves for differentially stored samples

Class	Chi-Square	Prob>Chi-Square
**Alphaproteobacteria**	3.85714	0.04953
**Deltaproteobacteria**	0.42857	0.51269
**Acidobacteria-6**	0.42857	0.51269
**Betaproteobacteria**	2.33333	0.12663
**Gammaproteobacteria**	0.04762	0.82726
**Bacilli**	2.33333	0.12663
**Thermoleophilia**	1.19048	0.27523
**[Spartobacteria]**	1.19048	0.27523
**Actinobacteria**	0.04762	0.82726
**Planctomycetia**	1.19048	0.27523
**Nitrospira**	1.19048	0.27523
**[Pedosphaerae]**	1.19048	0.27523
**Acidimicrobiia**	0.42857	0.51269
**[Saprospirae]**	1.19048	0.27523
**Cytophagia**	3.85714	0.04953
**[Chloracidobacteria]**	3.85714	0.04953
**Solibacteres**	1.19048	0.27523
**Anaerolineae**	0.42857	0.51269
**Acidobacteria-5**	2.33333	0.12663
**Clostridia**	0.04762	0.82726
**Sphingobacteriia**	2.33333	0.12663
**PAUC37f**	1.19048	0.27523
**Gemm-1**	0.42857	0.51269
**MB-A2-108**	3.85714	0.04953
**Gemmatimonadetes**	0.42857	0.51269
**PRR-12**	0.04762	0.82726
**RB25**	0.04762	0.82726
**Ellin6529**	1.19048	0.27523
**Unknown**	3.85714	0.04953

Table S6. The difference between DESS-stored and DNA/RNA Shield-stored microbial communities at the class level by Kruskall-Wallis ANOVA (blue - significant difference)

	Chi-Square	Prob>Chi-Square
**Bacillaceae**	1.19048	0.27523
**Unknown**	0.04762	0.82726
**Nitrososphaeraceae**	3.85714	0.04953
**Gaiellaceae**	1.19048	0.27523
**Cytophagaceae**	3.85714	0.04953
**Chitinophagaceae**	0.42857	0.51269
**Nitrospiraceae**	2.33333	0.12663
**Micrococcaceae**	1.19048	0.27523

**mb2424**	3.85714	0.04953
**0319-6A21**	0.04762	0.82726
**Planococcaceae**	0.42857	0.51269
**RB40**	3.85714	0.04953
**Ellin6075**	1.19048	0.27523
**Paenibacillaceae**	2.33333	0.12663
**Clostridiaceae**	0.04762	0.82726
**Nocardiaceae**	3.85714	0.04953
**Streptomycetaceae**	1.19048	0.27523
**EB1017**	3.85714	0.04953
**koll13**	3.85714	0.04953
**Nocardioidaceae**	1.19048	0.27523
**C111**	1.19048	0.27523

Table S7. The difference between -180 °C-stored and DNA/RNA Shield-stored microbial communities at the family level by Kruskall-Wallis ANOVA (blue - significant difference)

Family	Chi-Square	Prob>Chi-Square
**Bacillaceae**	2.33333	0.12663
**Unknown**	0.04762	0.82726
**Nitrososphaeraceae**	3.85714	0.04953
**Gaiellaceae**	2.33333	0.12663
**Cytophagaceae**	0.04762	0.82726
**Chitinophagaceae**	0.04762	0.82726
**Nitrospiraceae**	0.04762	0.82726
**Micrococcaceae**	3.85714	0.04953
**mb2424**	3.85714	0.04953
**0319-6A21**	0.42857	0.51269
**Planococcaceae**	2.33333	0.12663
**RB40**	0.42857	0.51269
**Ellin6075**	3.85714	0.04953
**Paenibacillaceae**	0.04762	0.82726
**Clostridiaceae**	0.04762	0.82726
**Nocardiaceae**	1.19048	0.27523
**Streptomycetaceae**	0.42857	0.51269
**EB1017**	0.04762	0.82726
**koll13**	3.85714	0.04953
**Nocardioidaceae**	3.85714	0.04953
**C111**	1.19048	0.27523

Table S8. The difference between DESS-stored and DNA/RNA Shield-stored microbial communities at the family level by Kruskall-Wallis ANOVA (blue - significant difference)

	No buffer -180°C	No buffer -20°C	No buffer 4°C	No buffer 23°C	DNA/RNA Shield -20°C	DNA/RNA Shield 4°C	DNA/RNA Shield 23°C	DESS -20°C	DESS 4°C	DESS 23°C
**No buffer -180**	1.00	0.96	0.95	0.95	0.95	0.90	0.98	0.88	0.94	0.94
**No buffer -20**	0.96	1.00	0.97	0.95	0.94	0.88	0.94	0.91	0.95	0.96
**No buffer 4**	0.95	0.97	1.00	0.96	0.96	0.92	0.94	0.89	0.94	0.98
**No buffer 23**	0.95	0.95	0.96	1.00	0.95	0.91	0.94	0.90	0.94	0.95
**DNA/RNA Shield -20**	0.95	0.94	0.96	0.95	1.00	0.95	0.98	0.87	0.94	0.96
**DNA/RNA Shield 4**	0.90	0.88	0.92	0.91	0.95	1.00	0.94	0.79	0.92	0.92
**DNA/RNA Shield 23**	0.98	0.94	0.94	0.94	0.98	0.94	1.00	0.86	0.95	0.94
**DESS -20**	0.88	0.91	0.89	0.90	0.87	0.79	0.86	1.00	0.90	0.91
**DESS 4**	0.94	0.95	0.94	0.94	0.94	0.92	0.95	0.90	1.00	0.97
**DESS 23**	0.94	0.96	0.98	0.95	0.96	0.92	0.94	0.91	0.97	1.00

Table S9. Spearman correlation between the differentially treated samples at the class taxonomic level ($\rho < 0.9$ is colored in red)

	No buffer -180°C	No buffer -20°C	No buffer 4°C	No buffer 23°C	DNA/RNA Shield -20°C	DNA/RNA Shield 4°C	DNA/RNA Shield 23°C	DESS -20°C	DESS 4°C	DESS 23°C
**No buffer -180**	1.00	0.60	0.85	0.94	0.93	0.92	0.96	0.86	0.93	0.89
**No buffer -20**	0.60	1.00	0.78	0.67	0.68	0.65	0.64	0.60	0.59	0.66
**No buffer 4**	0.85	0.78	1.00	0.93	0.90	0.89	0.87	0.81	0.83	0.91
**No buffer 23**	0.94	0.67	0.93	1.00	0.96	0.94	0.96	0.88	0.90	0.96
**DNA/RNA Shield -20**	0.93	0.68	0.90	0.96	1.00	0.93	0.97	0.89	0.89	0.93
**DNA/RNA Shield 4**	0.92	0.65	0.89	0.94	0.93	1.00	0.95	0.78	0.92	0.94
**DNA/RNA Shield 23**	0.96	0.64	0.87	0.96	0.97	0.95	1.00	0.88	0.92	0.92
**DESS -20**	0.86	0.60	0.81	0.88	0.89	0.78	0.88	1.00	0.87	0.89
**DESS 4**	0.93	0.59	0.83	0.90	0.89	0.92	0.92	0.87	1.00	0.94
**DESS 23**	0.89	0.66	0.91	0.96	0.93	0.94	0.92	0.89	0.94	1.00

Table S10. Spearman correlation between the differentially treated samples at the family taxonomic level ($\rho < 0.9$ is colored in red)

	No buffer -180°C	No buffer -20°C	No buffer 4°C	No buffer 23°C	DNA/RNA Shield -20°C	DNA/RNA Shield 4°C	DNA/RNA Shield 23°C	DESS -20°C	DESS 4°C	DESS 23°C

**No buffer -180**	1.00	0.61	0.64	0.57	0.75	0.64	0.75	0.73	0.65	0.73
**No buffer -20**	0.61	1.00	0.79	0.59	0.54	0.45	0.61	0.59	0.53	0.58
**No buffer 4**	0.64	0.79	1.00	0.69	0.62	0.56	0.62	0.57	0.53	0.64
**No buffer 23**	0.57	0.59	0.69	1.00	0.72	0.65	0.81	0.80	0.77	0.83
**DNA/RNA Shield -20**	0.75	0.54	0.62	0.72	1.00	0.76	0.86	0.87	0.78	0.77
**DNA/RNA Shield 4**	0.64	0.45	0.56	0.65	0.76	1.00	0.71	0.70	0.83	0.65
**DNA/RNA Shield 23**	0.75	0.61	0.62	0.81	0.86	0.71	1.00	0.85	0.85	0.77
**DESS -20**	0.73	0.59	0.57	0.80	0.87	0.70	0.85	1.00	0.85	0.76
**DESS 4**	0.65	0.53	0.53	0.77	0.78	0.83	0.85	0.85	1.00	0.74
**DESS 23**	0.73	0.58	0.64	0.83	0.77	0.65	0.77	0.76	0.74	1.00

Table S11. Spearman correlation between the differentially treated samples at the genus taxonomic level ($\rho < 0.6$ is colored in red)
